# Vegetarians’ and vegans’ experiences with and attitudes towards ultra-processed foods (UPF): a qualitative study

**DOI:** 10.1186/s40795-024-00925-y

**Published:** 2024-09-12

**Authors:** Johanne Haneberg, Marianne Molin, Marte Gjeitung Byfuglien, Lisa Garnweidner-Holme

**Affiliations:** 1 Department of Nursing and Health Promotion, Faculty of Health Sciences, Oslo Metropolitan University, St. Olavs Plass, P.O. Box 4, Oslo, 0130 Norway; 2 School of Health Sciences, Kristiania University College, PB 1190 Sentrum, Oslo, PB 0107 Norway; 3 Mills AS, Pb. 4644 Sofienberg, Oslo, 0506 Norway

**Keywords:** Ultra-processed foods, Vegetarian, Vegan, Health, Food waste, Sustainability, Qualitative research

## Abstract

**Background:**

The consumption of ultra-processed foods (UPF) is increasing in many countries. Simultaneously, there is a growing number of consumers that follow a vegetarian or vegan diet, many due to its possible positive impact on sustainability and food waste. However, little is known about attitudes towards and experiences with UPF among vegetarians and vegans. Thus, this study investigates vegetarians’ and vegans’ experiences with and attitudes towards UPFs.

**Methods:**

We conducted semi-structured, individual interviews with 14 participants between September and December 2021. The participants were from different areas in Norway. The data were analysed using a thematic analysis by Braun and Clarke.

**Results:**

In general, participants appeared to have diverse knowledge of and divergent attitudes towards UPFs. However, participants mainly associated substitute products (e.g. meat substitutes, dairy substitutes) as UPFs. They appreciated the increased availability of vegetarian and vegan UPF which made it easier for them to follow a plant-based diet. They enjoyed the taste and consistency of vegetarian and vegan UPF. However, participants expressed concerns about the effects that industrial processing has on the products’ nutritional content.

**Conclusion:**

This study indicated that there was a diverse knowledge of and various attitudes towards UPFs among the participating vegetarians and vegans. Public information and guidelines about using UPF (e.g. meat substitutes, dairy substitutes) in vegetarian and vegan diets are needed, as well as information about their possible impact on health and sustainability.

**Supplementary Information:**

The online version contains supplementary material available at 10.1186/s40795-024-00925-y.

## Introduction

There has been increased attention and a shift to more plant-based diets (e.g. vegetarian, vegan) worldwide [1]. The vegetarian diet mainly consists of plant-based foods (i.e. cereals, vegetables, fruits, berries, legumes, nuts and seeds), as the diet excludes meat. Whereas vegetarian diets are similar to vegan diets, the latter has the strictest dietary exclusion, avoiding all animal products, including eggs and dairy, and only eating plant-based foods [2]. Even if vegetarian or vegan diets are generally associated with beneficial health effects, this is dependent on the type of plant foods being consumed [3, 4]. People may choose a plant-based diet for different reasons and motivations (e.g. health, ethics, environmental, spiritual and religion) [5]. Among all motivations for vegetarians, priority is often attributed to perceived health benefits [6].

Simultaneously, with the increased number of vegetarians and vegans, there is a growing availability of industrial substitute products (e.g. meat substitutes, dairy substitutes), where a large share can be classified as being ultra-processed [7]. Ultra-processed foods (UPF) have become increasingly popular in both high- and middle-income countries [8, 9]. They are characteristically energy-dense, fatty, sugary or salty [8], and generally contain many added ingredients (e.g. sugar, salt, fat, artificial colours, preservatives). They are mostly made from substances extracted from foods (e.g. fats, starches, added sugars, hydrogenated fats) [10, 11]. The NOVA classification categorises all foods and food products into four groups according to the extent and purpose of the industrial processing they undergo, which are (1) unprocessed and minimally processed foods, (2) processed culinary ingredients, (3) processed foods and (4) UPF [12]. A high proportion of processed foods in general, including UPFs, have been associated with eating patterns and behaviours that might contribute to overweight, obesity and non-communicable diseases (NCDs) [12–16]. According to nationwide food surveys assessing intake, household expenses or supermarket sales, UPFs represent between 25% and 60% of total daily energy intake in European countries [17]. In Norway, a recent cross-sectional study of scanner data of food sales in grocery stores, found that the share of expenditure of UPFs was 46,5% in 2019 [18]. The NOVA classification has been criticised due to its too broad approach [19]. Previous research has also shown that the NOVA classification is confusing for consumers [20, 21]. An online study with 2,381 participants showed that most of the participants described UPFs as being highly-processed products that usually contain additives and other artificial ingredients, stressing that they have low nutritional quality and are unhealthy [22].

There is a growing consumption of UPFs by the population that follows a vegetarian or vegan diet. This is supported by findings from a previous study, concluding that the proportion of energy received from UPFs was significantly higher for vegetarians (37% of total energy intake) and vegans (39%) compared to meat-eaters (33%), being driven by a higher consumption of plant-based meat and dairy substitutes [7]. It is further discussed that a higher intake of the ultra-processed substitute products might reduce the potential health benefits of a plant-based diet, having the same risk of developing health problems as non-vegetarians [7]. However, 75% of non-vegetarians and people on plant-based diets have reported their self-perceived health as good or very good [23].

Despite emerging knowledge on the motivation for a vegetarian or vegan diet, less is known about vegetarians’ and vegans’ experiences with and attitudes towards UPFs. Therefore, we aim to gain more knowledge about this knowledge gap in the present study.

## Materials and methods

### Sampling and participants

We conducted individual interviews with 14 participants between September and December 2021. They were recruited based on the following inclusion criteria: defining themselves as a vegetarian/vegan, over 18 years old, primarily responsible for purchasing and cooking food in their home and have not studied and/or not studying nutrition. The participants were purposive recruited by spreading information about the study on the internet and social media (i.e. OsloMet website, closed groups for vegetarians and vegans on Facebook, and on social media of Mills AS). Participants themselves contacted the researchers. A snowball sampling was later used for further recruitment by asking the participants to spread information about the study to friends and family who fit the inclusion criteria.

### Data collection

We developed a semi-structured interview guide (see supplementary file 1). Some adjustments were made in the interview guide after being tested in the pilot interview. We ended up presenting the definition of UPFs in all the interviews, along with explaining general information, examples of UPFs, and the other food processing categories, as retrieved from the NOVA classification [12]. The final interview guide consisted of five main topics based on the study’s research questions (see supplementary file 1). As a result of Covid-19, all the interviews were conducted digitally over Zoom. The interviews were audio recorded using the application, Nettskjema-Diktafon [24]. Interviews were transcribed sequentially and verbatim by a member of the research team to preserve as much of the information as possible [25]. None of the participants asked to read the transcripts. The Norwegian Centre for Research Data (NSD: 950399) approved the study and it was conducted in accordance with both NSD and OsloMet’s ethical research guidelines. All participants gave informal, oral consent to be interviewed and for the interviews to be audio recorded and used for research purposes and publication. Our study was conducted in accordance with the Consolidated Criteria for Reporting Qualitative Research (COREQ) guidelines as a tool to report important aspects of the process [26].

### Analysis

The analysis was guided by thematic analysis [27]. Interviews were analysed inductively and consisted of six steps. First, the first author (JH) read through the transcripts from the interviews to become familiar with the data. Second, JH generated the initial codes based on phrases that were relevant to the study (e.g. related to vegetarians’ and vegans’ knowledge of UPFs). LGH and JH discussed the codes. Third, JH sorted different codes into potential sub-themes based on similarity, and then further into temporary main themes. Fourth, LGH reviewed the themes by overlooking and discussing them based on their relevance. Irrelevant themes were discharged. In addition, some new themes were created or combined. Fifth, JH, LGH and MM defined and redefined the themes. Sixth, JH wrote down the final result from the data. JH used NVivo (X9) to identify and organise the data into codes. All authors discussed the potential themes and sub-themes.

## Results

Table 1 provides the characteristics of the participants in our study. They varied in terms of gender, age, education, residence and years since committing to a vegetarian or vegan diet. 5 participants had been vegetarian/vegan for less than 5 years, 6 for less than 10 years and 3 were vegetarian for more than 10 years.

**Table 1:** Characteristics of the participants

Participant	Gender	Age	Education	Residence	Diet	Years being vegetarian or vegan diet	
1	Female	46–50	University	Rural	Vegetarian	10	
2	Male	26–30	University	Suburban	Vegetarian	10	
3	Female	36–40	University	Urban	Vegetarian	7	
4	Female	21–25	University	Urban	Vegetarian	6	
5	Female	51–55	University	Suburban	Vegetarian	9	
6	Male	41–45	Tertiary vocational education	Rural	Vegetarian	6	
7	Male	36–40	Upper secondary school	Rural	Vegan	4	
8	Female	21–25	University	Rural	Vegetarian	3	
9	Female	41–45	University	Rural	Vegetarian	9	
10	Male	31–35	Upper secondary school	Urban	Vegan	2	
11	Female	51–55	University	Urban	Vegetarian	2	
12	Female	21–25	Upper secondary school	Rural	Vegan	2	
13	Male	46–50	Tertiary vocational education	Suburban	Vegan	8	
14	Male	36–40	University	Urban	Vegetarian	14	

Table 2 summarises the main themes and sub-themes.

**Table 2:** Main themes and sub-themes

Main themes	Sub-themes
Experiences with UPFs	● Experience that UPFs work as an easy solution for eating vegan/vegetarian). ● Experience that they buy and eat UPFs in situations where there are no better alternatives. ● Experience that the selection of vegetarian and vegan food has improved.
Diverse knowledge of UPFs	● Had knowledge of UPFs before agreeing to participate in the study ● Had little or no knowledge of UPFs before agreeing to participate in the study ● Had knowledge of the existence of categorisation within the processing of food ● Had little or no knowledge about the existence of categorisation within the processing of food
Attitudes towards UPFs	● Wants to eat as little UPFs as possible, as they experience getting sick from it ● Wants to eat as little UPFs as possible, but finds it difficult to avoid ● Associates UPFs with something negative based on processing and content ● Believes that vegetarian and vegan UPFs make it easier for the consumer to eat more plant-based diet. ● Believes that vegetarian and vegan UPFs like legumes (such as lentils, beans, etc.) are good sources of protein. ● Believes that vegetarian and vegan UPFs can substitute animal products in both flavour and consistency. ● Believes that vegetarian and vegan UPFs are often processed. ● Believes that vegetarian and vegan UPFs are healthier than the comparable products of animal origin. ● Believes that UPFs can contribute negatively to food waste and sustainability based on processing and content. ● Believes that UPFs can contribute positively to food waste and sustainability based on processing and content.
Reasons for purchasing and/or eating UPFs	● Eats UPFs because of the taste. ● Eats UPFs because it is convenient. ● Eats UPFs more often when time is an issue. ● Eats UPFs more often in social contexts.
Increased awareness about food and sustainability because of vegetarianism and/or veganism	● Experienced an increased awareness of their own diet after becoming a vegetarian. ● Experienced an increased awareness regarding being a consumer after becoming a vegan.

### Experiences with UPFs

In general, it is important to mention that participants mainly perceived substitutes (e.g. meat substitutes, dairy substitutes) as UPFs. Several participants experienced availability and convenience as the main reasons for purchasing and eating UPFs. They experienced that these products work as an easy solution, in general, and in situations where there are no better alternatives. Even though time being vegan/vegetarian did not influence participantsæ experiences and attitudes towards UPFs’, especially participants who had been vegetarian for more than 10 years experienced the selection of vegetarian food improving, as illustrated by the following statement of a participant who had been vegetarian for 9 years:

“(…) I would rather not buy it, but sometimes they have no better alternative (…)” (Female, 41–45 years old, vegetarian).

All participants outlined that the increased availability of ultra-processed vegetarian and vegan food makes it easier to eat a plant-based diet overall. Some also mentioned that the majority of vegetarian and vegan UPFs are substitute products (e.g. meat and dairy replacements), which are processed to some extent:

“It has increased a lot in recent years. When I started eating vegetarian… About ten years ago, it was very difficult (…) It was much harder to get products before. Now I can go to almost any store, and I always find something” (Female, 46–50 years old, vegetarian for 10 years).

### Diverse knowledge of UPFs

We asked participants about their knowledge of UPF before agreeing to participate in the study. Some of the participants had little or no knowledge of UPFs, as exemplified in the following statement:

“No, I haven’t heard that expression” (Male, 36–40 years old, vegan for 4 years).

Also, participants who had been following a vegan/vegetarian diet for more than 5 years had little knowledge about UPFs. One participant who has been vegetarian for two years told that she just recently has heard about the term ultra-processed food in general: while for others, they learned about them in past years:

“Yes. I think. I don’t know exactly when the word appeared to me, but I haven’t known about it for too long. Maybe one or two years?” (Female, 21–25 years old, vegetarian).

Participants had sometimes heard about different categories of processing, primarily unprocessed, processed and ultra-processed.

### Attitudes towards UPFs

Participants had divergent attitudes towards UPFs. Negative attitudes were related to industrial processing and the products’ nutritional content. One participant believed that industrial processing could cause negative health consequences due to overeating and obesity. Other participants believed that UPFs, in general, were processed to such an extent that it is hard to recognise the raw materials on which they are based.

“(…) So the products are processed in a way that it’s hard to recognize the raw materials they’re based on (…)” (Female, 51–55 years old, vegetarian for two years).

Several participants mentioned different reasons for avoiding UPFs. One participant said that she was feeling sick from eating too much UPFs, while another said that it was hard to avoid in a community where most of the available food was ultra-processed:

“(…) It’s not something we eat a lot of, but it’s almost impossible to avoid in today’s society where a lot is ultra-processed” (Female, 36–40 years old, vegetarian for 8 years).

The participants who had a more positive attitude towards UPFs acknowledged that vegetarian and vegan UPFs made it easier for them to eat more plant-based foods. Other participants mentioned how vegetarian and vegan UPFs based on legumes functioned as a good source of protein, and overall, might be healthier than comparable products of animal origin:

“(…) I think it’s very good that there are easy ways to do it, especially for those who don’t have much knowledge and can just go to the store and find something simple and fast (…) I’m glad that there are offers that make it easy for everyone to choose (…)” (Male, 36–40 years old, vegan for 4 years).

Others, independent of how long they have followed a vegan/vegetarian diet, enjoyed the taste and consistency of vegetarian and vegan UPFs. They also reflected on how UPFs may affect food waste and sustainability, both positively and negatively. Some questioned how sustainable the content and processing of these products were:

“(…) They send the food across land and oceans. And when a product is that processed and consists of that many ingredients, I doubt that all the ingredients and processes are done locally (…)” (Female, 46–50 years old, vegetarian for more than 10 years).

According to the remaining participants, UPFs could contribute positively to the shelf life of these foods in comparison to whole foods, which potentially could reduce food waste:

“(…) But in relation to that, one would think that UPFs will result in less food waste than just fresh ingredients and that because the store always is dependent on the fresh ingredients being sold (…) You don’t have to throw away UPFs as often. They have a long shelf life (…)” (Male, 31–35 years old, vegan for two years).

### Reasons for buying and/or eating UPFs

The participants mentioned different reasons for buying and/or eating UPFs independently on how long the have been vegan/vegetarian. Some bought and ate UPFs owned to good taste and convenience. Others had the impression that the general consumer mainly chooses UPFs because they prefer the given consistency and taste and because they are familiar with this type of food. A participant confirmed this:

“(…) And because it tastes good. It reminds me of chicken, as an example, which is a flavour I don’t get otherwise. There are a lot of legumes and tofu in the vegetarian diet, and I don’t think it’s similar to the products I do miss (…)” (Female, 21–25 years old, vegetarian for 6 years).

Other participants mentioned that they were eating UPFs more often in certain situations, for example, in social contexts and when time is an issue:

“I think it might be when we’re going out to eat and so on… If I’m going out to eat with friends. We often go out to eat somewhere where everyone can find something, even though I preferably would avoid it. But it seems to me that most of the people like the most processed foods (…)” (Female, 21–25 years old, vegetarian for 3 years).

### Increased awareness about sustainability because of vegetarianism and/or veganism

Especially participants who recently (< 3 years) had become vegan/vegetarian were concerned about the sustainability of UPF. They reflected upon how they gained an increased awareness about sustainability because of vegetarianism or veganism. Some experienced an increased awareness of their own diet and the effects of being a consumer:

“(…) Also, before I turned vegan, but I must admit that veganism has affected many aspects of my life. For example, recycling as much as possible, using as little plastic as possible in general, not buying things I don’t need and so on (…)” (Male, 31–35 years old, vegan for two years).

## Discussion

Participants in this study appeared to have diverse knowledge of and attitudes towards UPFs. In discussing our results, we focus on UPFs in the form of substitute products (e.g. meat substitutes, dairy substitutes), because participants mainly mentioned these types of products when we asked them what they perceived as UPFs. In line with previous research among omnivore consumers [22], most participants had heard about different categories of processing foods, primarily unprocessed, processed and ultra-processed. For a few participants, the presentation on categorising UPFs was new. In line with previous research, a practical and simple way to identify UPFs is by searching for ingredients and additives that are not commonly used in kitchens (e.g. high-fructose corn syrup, hydrolysed proteins) or classes of additives designed to make the final product palatable or more appealing (e.g. flavours, colours, thickeners) [28].

For several participants, availability and convenience were the main reasons to purchase and eat UPFs. A cross-national study looking at the perceived importance of food choice motives found that convenience and taste were some of the main reasons underlying food choices [29]. On the other hand, there has been a significant decrease in consumers who say that preparing food needs to be quick and simple, and an increasing number who say that they value fresh, unprocessed ingredients [30]. Hence, it appears to be important to provide vegetarians and vegans with knowledge about how to easily prepare meals with whole foods and minimally processed foods.

All participants felt that the selection of vegetarian and vegan food had improved and that the rise in both selection and availability made it easier to eat a plant-based diet overall. In line with the total increased consumption of UPFs [8, 17], there has been a growing interest in plant-based substitute products [31–33]. As a result, the availability and convenience of these products have improved [31, 32, 34]. The availability of vegetarian food products is one of the factors that help vegetarians and vegans maintain their diets [35]. Varela et al. [36] found that many consumers perceive industrial products (e.g. meat and dairy substitutes) as highly processed. Although plant-based substitutes may provide a higher intake of recommended food groups, the nutritional components of the products on the market vary [37], and so a large amount of these can be classified as ultra-processed [7]. This is in line with a French cross-sectional study by Gehring et al. [7], who found that a sample of vegetarians and vegans took a higher proportion of energy from UPFs than meat-eaters, where the consumption of plant-based substitutes played a part [7].

Participants had divergent attitudes towards UPFs. Negative attitudes were related to industrial processing and the products’ nutritional content. In general, UPFs have unfavourable nutrient profiles and several other characteristics linked to negative health outcomes [11, 13, 14, 38, 39]. An American study found that the ultra-processed diet caused increased ad libitum (i.e. as much or as often as desired) energy intake and weight gain. This was even though the diets were matched for presented calories, sugar, fat, fibre and macronutrients [40]. In contrast to these studies, we have to consider that our participants were asked about vegetarian and vegan UPFs, the content of which might be more nutritious than meat-based UPFs.

Some participants mentioned different reasons for avoiding eating UPFs. Participants mentioned that they were feeling sick from eating too much of it and that it was hard to avoid in a community where most of the food available was ultra-processed. A series of major differences between vegetarians’ and vegans’ food and eating behaviours based on individuals’ motivations (e.g. health, ethics, environment) has previously been identified [35]. A Canadian survey examining food choices, motivations and dietary identity [41] found that, among the group unlikely to purchase meat substitutes, the main reasons were the food being “too processed” and “high in sodium” [42]. Different motivations can result in different food choices; hence, it would be wrong to assume that they all are heavy users of substitute products and UPFs, in general.

Participants believed that vegetarian and vegan UPFs made it easier for the average consumer to eat more plant-based foods. Looking into millennials’ consumption of and attitudes toward plant-based meat alternatives in Finland, Knaapila, Michel and colleagues [43] found that women on average had more positive associations with plant-based substitute products than men. Further, the study confirmed that vegetarians and vegans reported positive associations with meat substitutes more frequently than others [43].

For some participants, taste and consistency were the main reasons for purchasing and eating vegetarian and vegan UPFs. Gehring et al. [7] found that people who recently started a vegetarian or vegan diet were more likely to eat more UPFs than those who became a vegetarian or vegan long ago. We did not find that the duration of being vegan/vegetarian did influence participants experiences and attitudes towards UPFs. Also, in this study, participants valued the consistency and taste of vegetarian and vegan UPF which was similar to meat-based products [7]. This is supported by a Dutch study by Hoek et al. [44] that investigated food-related lifestyle and health and found that vegetarians and vegans use substitute products as a way to abandon animal products [44]. Animal products play an important role in many people’s food cultures, and plant-based substitute products can therefore make it easier to begin a vegetarian or vegan diet [3].

Among the participants, there were also perceptions regarding how UPFs affect food waste and sustainability related to climate change. Some participants questioned how sustainable the content and processing of UPFs was, while others believed that these products could potentially reduce food waste based on their shelf life in comparison to whole foods. According to a cross-sectional qualitative survey investigating consumers’ perceptions of healthy and sustainable diets by Van Loo et al. [45], a plant-based diet is, in general, associated with being “healthy” and “sustainable” [45]. Marketing strategies for plant-based substitute products take advantage of this by highlighting the nutritional benefits of the plant-based ingredients in the product [7]. Varela et al. [36] found that many participants seem to find a conflict between health and sustainability in these products, particularly meat and dairy substitutes [36]. Industrial processing is discussed regarding its harm to the environment [12]. Still, some are relating the industrial processing of UPFs with shelf life extension [22].

Some participants mentioned that they were eating UPFs more often in certain situations (e.g. in social contexts and when time is an issue). Concerning social contexts, Hoek et al.[44] found that vegetarians seem to prefer eating with friends [44] and might, as a result, be more open to consuming UPFs in social contexts. In addition, a British study evaluating the association between eating context patterns and consuming UPFs [46] found that eating with friends in food outlets or at a friend’s house was associated with higher daily consumption of UPFs. A previous British study supports this finding, reporting that energy intake increased by 18% when eating with friends [47]. Issues related to time are often taken into account as a criterion for convenience. People nowadays are generally searching for convenience foods more often and may be more likely to consume less healthy foods, such as substitute products that are ultra-processed [48].

In our study, especially participants who had been vegan/vegetarian for less than five years were concerned about the sustainability of UPF. According to a Norwegian study on vegetarianism [2], the most common reason (71%) for having a vegetarian diet was the positive effects on the climate and environment. This is supported by a Korean review on meat analogues as a future food [49], reflecting on how the most significant interest in substitute products is not due to the increased number of vegetarians and vegans, but is driven by the consumers concerned about healthy foods and a sustainable environment. Clark et al. estimated the environmental impact of 57,000 food products [50]. The study shows a tendency for more nutritious foods to be more environmentally sustainable, and that like-for-like substitutes can have highly variable environmental and nutritional impacts [50]. As many consumers find a conflict between health and sustainability in UPFs, the exposure to more and new products based on legumes, grains and cereals, not based on imitating meat, could promote the transition to more sustainable and healthier diets [36].

### Study limitations

This study was conducted with a small sample size, which is typical of qualitative studies [51]. The study focused on vegans/vegetarians experiences with UPM, hence, results cannot be compared with similar studies among omnivores. Recruitment via the Internet and social media might have included participants who were already interested in UPF. As a result of Covid-19, all interviews were conducted via Zoom, which might have affected the natural communication between the participant and the interviewer.

## Conclusion

This study indicated that there was a diverse knowledge of and various attitudes towards UPFs among the participating vegetarians and vegans. Public information and guidelines about using UPF in vegetarian and vegan diets are needed, as well as information about their possible impact on health and sustainability.

## Supplementary Information

Supplementary Material 1.

## Data Availability

Data will be made available upon request from the authors.

## Abbreviations

COREQ: Consolidated Criteria for Reporting Qualitative Research
NSD: Norwegian Centre for Research Data
UPF: Ultra-processed food

## References

[1] World Health Organization. Plant-based diets and their impact on health, sustainability and the environment: a review of the evidence. WHO European Office for the Prevention and Control of Noncommunicable Diseases; 2021.

[2] Bugge AB, Henjum S. Vegetarianisme–en studie av sosiale, praktiske og kroppslige aspekt ved å ha et helt eller delvis vegetarisk spisemønster. SIFO-rapport nr. 4-2021 (oda.oslomet.no). Oslo: Forbruksforskningsinstituttet SIFO, OsloMet; 2021.

[3] Salomé M, Huneau J-F, Le Baron C, Kesse-Guyot E, Fouillet H, Mariotti F. Substituting meat or dairy products with plant-based substitutes has small and heterogeneous effects on diet quality and nutrient security: a simulation study in French adults (INCA3). J Nutr. 2021;151(8):2435–45. 34049399 10.1093/jn/nxab146

[4] Satija A, Bhupathiraju SN, Spiegelman D, Chiuve SE, Manson JE, Willett W, et al. Healthful and unhealthful plant-based diets and the risk of coronary heart disease in US adults. J Am Coll Cardiol. 2017;70(4):411–22. 28728684 10.1016/j.jacc.2017.05.047 PMC5555375

[5] Petti A, Palmieri B, Vadala’ M, Laurino C. Vegetarianism and veganism: not only benefits but also gaps. A review. Progress Nutr. 2017;19:229–42.

[6] Fehér A, Gazdecki M, Véha M, Szakály M, Szakály Z. A Comprehensive Review of the benefits of and the barriers to the switch to a plant-based Diet. Sustainability. 2020;12(10):4136. 10.3390/su12104136

[7] Gehring J, Touvier M, Baudry J, Julia C, Buscail C, Srour B, et al. Consumption of ultra-processed foods by Pesco-vegetarians, vegetarians, and vegans: associations with duration and age at diet initiation. J Nutr. 2021;151(1):120–31. 32692345 10.1093/jn/nxaa196

[8] Monteiro CA, Moubarac JC, Cannon G, Ng SW, Popkin B. Ultra-processed products are becoming dominant in the global food system. Obes Rev. 2013;14(Suppl 2):21–8. 24102801 10.1111/obr.12107

[9] Alcorta A, Porta A, Tárrega A, Alvarez MD, Vaquero MP. Foods for plant-based diets: challenges and innovations. Foods. 2021;10(2):293. 33535684 10.3390/foods10020293 PMC7912826

[10] Tavares GA, Werneck A, Martins C, Tramontt C, Neri D, Silva E, et al. Dialogue on: ultra-processed food products: solutions for healthy and sustainable food systems. Cátedra Josué de Castro, Nupens USP; 2021.

[11] Ludwig DS. Technology, diet, and the burden of chronic disease. JAMA. 2011;305(13):1352–3. 21467290 10.1001/jama.2011.380

[12] Monteiro CA, Cannon G, Lawrence M, Costa Louzada M, Pereira Machado P. Ultra-processed foods, diet quality, and health using the NOVA classification system. Rome: FAO; 2019. p. 48.

[13] Bonaccio M, Di Castelnuovo A, Ruggiero E, Costanzo S, Grosso G, De Curtis A, et al. Joint association of food nutritional profile by nutri-score front-of-pack label and ultra-processed food intake with mortality: Moli-Sani prospective cohort study. BMJ. 2022;378:e070688. 36450651 10.1136/bmj-2022-070688 PMC9430377

[14] Wang L, Du M, Wang K, Khandpur N, Rossato SL, Drouin-Chartier J-P, et al. Association of ultra-processed food consumption with colorectal cancer risk among men and women: results from three prospective US cohort studies. BMJ. 2022;378:e068921. 38752573 10.1136/bmj-2021-068921 PMC9430376

[15] Dicken SJ, Batterham RL. Ultra-processed food: a global problem requiring a global solution. Lancet Diabetes Endocrinol. 2022;10(10):691–4. 36037821 10.1016/S2213-8587(22)00248-0

[16] Dicken SJ, Batterham RL. The role of diet quality in mediating the association between ultra-processed food intake, obesity and health-related outcomes: a review of prospective cohort studies. Nutrients. 2022;14(1):23. 10.3390/nu14010023 PMC8747015 35010898

[17] Srour B, Fezeu LK, Kesse-Guyot E, Allès B, Méjean C, Andrianasolo RM et al. Ultra-processed food intake and risk of cardiovascular disease: prospective cohort study (NutriNet-Santé). BMJ. 2019;365:l1451. 31142457 10.1136/bmj.l1451 PMC6538975

[18] Bjøntegaard MM, Kolby M, Molin M, Torheim LE. Purchase of ultra-processed foods in Norway: a repeated cross-sectional analysis of food sales in 2013 and 2019. Public Health Nutr. 2023;26(9):1743–53. 37339927 10.1017/S1368980023001192 PMC10478042

[19] Gibney MJ, Forde CG, Mullally D, Gibney ER. Ultra-processed foods in human health: a critical appraisal. Am J Clin Nutr. 2017;106(3):717–24. 28793996 10.3945/ajcn.117.160440

[20] Hässig A, Hartmann C, Sanchez-Siles L, Siegrist M. Perceived degree of food processing as a cue for perceived healthiness: the NOVA system mirrors consumers’ perceptions. Food Qual Prefer. 2023;110:104944. 10.1016/j.foodqual.2023.104944

[21] Sanchez-Siles L, Roman S, Fogliano V, Siegrist M. Naturalness and healthiness in ultra-processed foods: a multidisciplinary perspective and case study. Trends Food Sci Technol. 2022;129:667–73. 10.1016/j.tifs.2022.11.009

[22] Ares G, Vidal L, Allegue G, Giménez A, Bandeira E, Moratorio X, et al. Consumers’ conceptualization of ultra-processed foods. Appetite. 2016;105:611–7. 27349706 10.1016/j.appet.2016.06.028

[23] Groufh-Jacobsen S, Bahr Bugge A, Morseth M, Pedersen JT, Henjum S. Dietary habits and self-reported health measures among Norwegian adults adhering to plant-based diets. Front Nutr. 2022;824:1–9. 10.3389/fnut.2022.813482 PMC9094011 35571900

[24] University of Oslo. Nettskjema-dictaphone 2019 https://www.uio.no/english/services/it/adm-services/nettskjema/help/nettskjema-dictaphone.html

[25] Thagaard T. Systematikk Og innlevelse. En innføring i kvalitativ metode. Fagbokforlaget Vigmostad & Bjørke AS; 2013. p. 222.

[26] Tong A, Sainsbury P, Craig J. Consolidated criteria for reporting qualitative research (COREQ): a 32-item checklist for interviews and focus groups. Int J Qual Health Care. 2007;19(6):349–57. 17872937 10.1093/intqhc/mzm042

[27] Braun V, Clarke V. Using thematic analysis in psychology. Qualitative Res Psychol. 2006;3(2):77–101. 10.1191/1478088706qp063oa

[28] Monteiro CA, Cannon G, Levy RB, Moubarac J-C, Louzada ML, Rauber F, et al. Ultra-processed foods: what they are and how to identify them. Public Health Nutr. 2019;22(5):936–41. 30744710 10.1017/S1368980018003762 PMC10260459

[29] Markovina J, Stewart-Knox BJ, Rankin A, Gibney M, de Almeida MDV, Fischer A, et al. Food4Me study: validity and reliability of food choice questionnaire in 9 European countries. Food Qual Prefer. 2015;45:26–32. 10.1016/j.foodqual.2015.05.002

[30] Bugge AB. Mat, måltid og moral-hvordan spise rett og riktig? 2015. https://www.semanticscholar.org/paper/Mat%2C-m%C3%A5ltid-og-moral-hvordan-spise-rett-og-riktig-Bugge/0e94589b59d240122f8931189de688c50a0afd8e.

[31] Flere nordmenn. velger plantebasert mat [press release]. 14.12.2020 2020.

[32] Ipsos. 4 av 10 vil prøve plantebaserte kjøtterstatninger 2019 [updated 02.10.2019. https://www.ipsos.com/nb-no/4-av-10-vil-prove-plantebaserte-kjotterstatninger

[33] Costa IM, Barbosa CD, da Silva NH. Plant-based products: pontential, production technology and challenges. J Eng Exact Sci. 2022;8:14788. 10.18540/jcecvl8iss7pp14788-01e

[34] Grundekjøn C. Vegetarboom i Norge: Kjedene Kjemper Om «morgendagens kunder». E24. 2021 02.01.2021.

[35] Ruby MB. Vegetarianism. A blossoming field of study. Appetite. 2012;58(1):141–50. 22001025 10.1016/j.appet.2011.09.019

[36] Varela P, Arvisenet G, Gonera A, Myhrer KS, Fifi V, Valentin D. Meat replacer? No thanks! The clash between naturalness and processing: an explorative study of the perception of plant-based foods. Appetite. 2022;169:105793. 34748877 10.1016/j.appet.2021.105793

[37] Tonheim LE. Dietary intake and consumption of plant-based substitutes in vegans, vegetarians and pescatarians in Norway. OsloMet-storbyuniversitetet; 2021.

[38] Monteiro CA. Nutrition and health. The issue is not food, nor nutrients, so much as processing. Public Health Nutr. 2009;12(5):729–31. 19366466 10.1017/S1368980009005291

[39] Moodie R, Stuckler D, Monteiro C, Sheron N, Neal B, Thamarangsi T, et al. Profits and pandemics: prevention of harmful effects of tobacco, alcohol, and ultra-processed food and drink industries. Lancet. 2013;381(9867):670–9. 23410611 10.1016/S0140-6736(12)62089-3

[40] Hall KD, Ayuketah A, Brychta R, Cai H, Cassimatis T, Chen KY, et al. Ultra-processed diets cause excess calorie intake and weight gain: an inpatient randomized controlled trial of ad libitum food intake. Cell Metab. 2019;30(1):67-e773. 31105044 10.1016/j.cmet.2019.05.008 PMC7946062

[41] Clark LF, Bogdan A-M. The role of plant-based foods in Canadian diets: a survey examining food choices, motivations and dietary identity. J food Prod Mark. 2019;25(4):355–77. 10.1080/10454446.2019.1566806

[42] Machín L, Cabrera M, Curutchet MR, Martínez J, Giménez A, Ares G. Consumer perception of the healthfulness of ultra-processed products featuring different front-of-pack nutrition labeling schemes. J Nutr Educ Behav. 2017;49(4):330–8. e1. 28185813 10.1016/j.jneb.2016.12.003

[43] Knaapila A, Michel F, Jouppila K, Sontag-Strohm T, Piironen V. Millennials’ consumption of and attitudes toward meat and plant-based meat alternatives by consumer segment in Finland. Foods. 2022;11(3):456. 35159606 10.3390/foods11030456 PMC8834568

[44] Hoek AC, Luning PA, Stafleu A, de Graaf C. Food-related lifestyle and health attitudes of Dutch vegetarians, non-vegetarian consumers of meat substitutes, and meat consumers. Appetite. 2004;42(3):265–72. 15183917 10.1016/j.appet.2003.12.003

[45] Van Loo EJ, Hoefkens C, Verbeke W. Healthy, sustainable and plant-based eating: Perceived (mis) match and involvement-based consumer segments as targets for future policy. Food Policy. 2017;69:46–57. 10.1016/j.foodpol.2017.03.001

[46] Rauber F, Martins C, Azeredo C, Leffa P, Louzada M, Levy R. Eating context and ultraprocessed food consumption among UK adolescents. Br J Nutr. 2022;127(1):112–22. 33691816 10.1017/S0007114521000854

[47] Hetherington MM, Anderson AS, Norton GN, Newson L. Situational effects on meal intake: a comparison of eating alone and eating with others. Physiol Behav. 2006;88(4–5):498–505. 16757007 10.1016/j.physbeh.2006.04.025

[48] Peltner J, Thiele S. Convenience-based food purchase patterns: identification and associations with dietary quality, sociodemographic factors and attitudes. Public Health Nutr. 2018;21(3):558–70. 29173221 10.1017/S1368980017003378 PMC10261000

[49] Ismail I, Hwang Y-H, Joo S-T. Meat analog as future food: a review. J Anim Sci Technol. 2020;62(2):111. 32292920 10.5187/jast.2020.62.2.111 PMC7142285

[50] Clark M, Springmann M, Rayner M, Scarborough P, Hill J, Tilman D, et al. Estimating the environmental impacts of 57,000 food products. Proc Natl Acad Sci U S A. 2022;119(33):e2120584119. 35939701 10.1073/pnas.2120584119 PMC9388151

[51] Crouch M, McKenzie H. The logic of small samples in interview-based qualitative research. Social Sci Inform. 2006;45(4):483–99. 10.1177/0539018406069584

