# Supplementary material for: Vegetarians’ and vegans’ experiences with and attitudes towards ultra-processed foods (UPF): a qualitative study
Source: BMC Nutr. 2024 Sep 12;10:121. doi: 10.1186/s40795-024-00925-y (PMC11396134; doi:10.1186/s40795-024-00925-y)
Supplement: Supplementary file 1 — Supplementary Material 1. [file 40795_2024_925_MOESM1_ESM.docx]

**BEFORE THE INTERVIEW**

**Welcome the participant and thank them for participating.**

My name is Johanne.

I have a bachelor's degree in Public Health Nutrition from OsloMet, and now I'm in the process of finishing my master's.

The research you have chosen to participate in today is my master thesis, and I feel lucky to be able to work with something I find interesting.

When it comes to the purpose of this research, it is to gain a better insight into vegetarians' knowledge of and perceptions of ultra-processed food.

**Do you have previous experience participating in any research?**

Before I go a bit further into how we should proceed I want to check that you meet the inclusion criteria I have set for the study:

- Define yourself as a vegetarian?
- Over 18 years old?
- Is the main person responsible for shopping and cooking food in your household?
- Have not studied or are studying nutrition?

**Good! Now I want to go further into how we'll conduct the interview:**

The whole process will take 30-60 minutes. I will be asking you questions, and you'll answer as best you can. The interview itself will be recorded to be able to work with the information correctly in retrospect.

In this research, we collect yellow data, which means that we cannot access sensitive data (e.g., related to our own health).

During the actual interview, I will stay neutral to get the most realistic answers possible.

At the end of the interview, I will collect general information about you (e.g., age, gender, place of residence, education).

In the published master's thesis or any scientific publications, there will be no kind of information that is directly linked to you.

**Now I will briefly inform you a little about privacy.**

When it comes to privacy, the information is treated confidentially and in accordance with the privacy regulations.

It will only be myself and my supervisors who will have access to your personally identifiable information.

You have the right to inspect what is registered and to make any changes during the entire process. In addition, you can withdraw from the study at any time without giving a reason.

**Is there something that is unclear at this point, or do you have any questions?**

We'll be starting the interview very soon. First, I would like to assure that you've read the information letter and the consent letter so that you are informed about the purpose of the interview?

As we conduct the interview digitally and want to make it easy for the participant, I would like to ask for oral consent.

If you are ready, I will immediately start the audio recording and read up the consent.

Do you agree to participate in a research interview on Zoom in relation to our research, and that the interview itself will be audio-recorded in an independent tool called Nettskjema-Diktafon?

YES!

I have some questions to start with:

| **Main questions** | **Possible follow-up** |
| --- | --- |
| **1) Opening questions** | |
| How long have you been a vegetarian? |  |
| Would you like to tell me why you chose a vegetarian diet?  *(If they have not been all their life)* |  |
| What are your general experiences with the selection of vegetarian food? | How has the selection changed in recent years? |
| **2) Theme 1: Knowledge of ultra-processed foods (UPF)** | |
| Had you heard of ultra-processed foods before agreeing to join our study? |  |
| Have you seen the recent debate in the media regarding vegetarian ultra-processed foods? |  |
| What do you associate with the term "ultra-processed foods"? |  |
| Have you heard that there are different categories for processed foods (i.e., to what extent they are processed)? |  |

**To ensure that we have a common understanding of the concept of ultra-processed food, I will now give a brief introduction to what ultra-processed food is and the different categories of processing.**

Ultra-processed foods are pure industrial products that are not similar to the raw materials they're based on. They often have a high content of ingredients that are not found in a regular kitchen, such as consistency, thickeners, flavor enhancers, and dyes.

As I mentioned, foods can be divided into categories according to the degree of processing, where one goes from the lowest degree of processing up to the highest.

The first category

- E.g., vegetables, fruits, eggs, nuts, and milk
- These are raw materials directly from nature or raw materials that have been cleaned, dried, grounded, heat-treated, or frozen
- This category is unprocessed and minimally processed food

The second category

- E.g., sugar, vegetable oils, and sifted flour
- These are raw materials that have been pressed, refined, grounded, or freeze-dried, and will further be used in the production of food products
- This category is processed culinary ingredients

The third category

- E.g., smoked meat, pickled fruit, canned fish, and various cheeses
- These are raw materials that are boiled, fried, fermented, or only added with certain additives such as salt, sugar, or oil
- This category is processed foods

The fourth and final cate gory

- E.g., potato chips, energy bars, ice cream, frozen pizza, biscuits, and various products made from leftovers from fish and meat
- In this category, the foods are largely composed of ingredients as opposed to raw materials. Here we can find a mixture of vegetable oils, starch, flavorings, dyers, emulsifiers, and consistency additives. In other words: ingredients not found in a regular kitchen
- This is what we know as ultra-processed foods

**Did you get an understanding of what ultra-processed food is?**

Then we move on with questions.

| **3) Theme 2: What perception do vegetarians have about UPFs?** | |
| --- | --- |
| What is your general perception of ultra-processed foods? |  |
| Do you usually buy and/or eat ultra-processed foods? | If so, why do you buy and/or eat ultra-processed foods? |
| Which meals and possibly in which situations do you most often eat ultra-processed foods? |  |
| Which products of those categorized as ultra-processed are your favorites? |  |
| **4) Theme 3: What is vegetarians' perception of UPFs and health?** | |
| What do you think a healthy diet should consist of? |  |
| What is your opinion of the nutritional quality of vegetarian food that is ultra-processed compared to less processed foods? |  |

| **5) Theme 4: What are vegetarians' perceptions about UPFs connection to food waste?** | |
| --- | --- |
| How do you deal with food waste? | What are you doing to reduce food waste? |
| Do you have any thoughts on ultra-processed food and how it relates to food waste? |  |
| **6) Theme 5: What are vegetarians' perceptions of UPF related to sustainability?** | |
| Are you concerned about whether food is sustainable when you 're choosing food? | Do you take it into account when choosing what to eat? |
| Do you have any thoughts on ultra-processed food and how such foods belong in a sustainable diet? |  |
| **7) Closing questions** | |
| If you are going to reflect on ultra-processed foods all over again now, what do you think? |  |
| Is there anything you want to add that I have not asked? |  |
| **8) Background questions** | |
| Age? |  |
| Gender? |  |
| Marital status/do you live with someone? |  |
| Residence? |  |
| Highest achieved education? |  |
| Where did you hear about this research? |  |
| The interview was conducted digitally/physically |  |
